# The Effectiveness of the Headspace App for Improving Sleep: Randomized Controlled Trial

**DOI:** 10.2196/56287

**Published:** 2026-02-04

**Authors:** Zoltan Andre Torok, Larisa Gavrilova, Amish Patel, Matthew Jason Zawadzki

**Affiliations:** 1 Department of Psychological Sciences, University of California, 5200 North Lake Road, Merced, CA, 95343, United States, 1 5598712655

**Keywords:** Headspace, sleep quality, tiredness, sleep duration, sleep efficiency, ecological momentary assessment, app-based, sleep, subjective, objective, outcome, meditation, randomized controlled trial, RCT, application, self-guided, female, women, United States, mHealth, mobile health, Fitbit, wearable

## Abstract

**Background:**

Improving sleep is critical for optimizing short-term and long-term health. Although in-person meditation training has been shown to impact sleep positively, there is a gap in our understanding of whether apps that teach self-guided meditation are also effective.

**Objective:**

This study aims to test whether Headspace (Headspace, Inc) improves sleep quality, tiredness, sleep duration, and sleep efficiency.

**Methods:**

Staff employees (N=135; mean age 38.1, SD 10.9; 75.0% female; 59.3% non-Hispanic White; 27.1% Hispanic) from a university in California’s San Joaquin Valley participated in the study. Participants were randomized to complete 10 minutes of daily meditation via the Headspace app for 8 weeks or waitlist control. Sleep assessments were taken for 4 consecutive days at baseline, and then for 4-day bursts at 2, 5, and 8 weeks after randomization. Sleep quality and subjective sleep duration were assessed each morning with a sleep diary, tiredness was assessed throughout the day using ecological momentary assessment, and objective sleep duration and efficiency were measured using a Fitbit Charge 2.

**Results:**

Both subjective and objective sleep outcomes improved. For subjective sleep outcomes, multilevel modeling revealed that those in the Headspace condition, compared to the control group, reported better sleep quality at sessions 2 (β=0.48, SE=0.12; *P*<.001), 5 (β=0.91, SE=0.13; *P*<.001), and 8 (β=0.69, SE=0.15; *P*<.001) compared to baseline, and a decrease in tiredness at session 5 (β=−0.58, SE=0.19; *P*=.001) compared to baseline, but not at sessions 2 or 8. For objective sleep outcomes, those in the Headspace condition compared to the control group had longer sleep durations at session 5 (β=23.96, SE=12.19; *P*=.04) compared to baseline, but not at sessions 2 or 8. There were no significant effects for sleep efficiency.

**Conclusions:**

This study continues adding to the ever-developing field of mobile health apps by demonstrating that Headspace can positively impact sleep quality, tiredness, and duration.

## Introduction

### Overview

Training in mindfulness meditation has shown the ability to improve sleep outcomes, such as sleep quality and efficiency [1,2]. Most of this research has tested meditation as traditional in-person mindfulness meditation practices, including mindfulness-based stress reduction and mindfulness-based therapy for insomnia [3,4]. There has been a shift in recent years toward using mobile health (mHealth) apps, such as Headspace (Headspace, Inc) and Calm (Calm.com, Inc), to learn meditation and mindfulness. Therefore, because of their increased popularity, these apps are critical to study as users are seeking these apps to improve their health, including sleep [5]. In addition, they have the potential to be more accessible and available than in-person practices. However, these benefits may come with a trade-off, as these apps are generally self-guided and may not be as effective in teaching meditation or mindfulness practices. App designers often focus on usability and acceptability rather than effectiveness in improving health outcomes [6]. Thus, it is necessary to rigorously examine whether mHealth apps for meditation can effectively improve sleep outcomes. Furthermore, there is a need to research when these positive sleep effects emerge, enabling the ability to track the efficacy of interventions during engagement rather than waiting until a postintervention evaluation period.

### Sleep Is a Complex Construct

Sleep is a complex, multidimensional construct that can be measured subjectively and objectively. A well-supported approach to studying sleep identifies 5 dimensions: sleep quality, sleep duration, sleep continuity, sleep timing, and sleepiness [7]. Given that each dimension of sleep can change independently of the others depending on the intervention, it is advisable to measure multiple dimensions. Subjective sleep is self-reported and evaluated using retrospective surveys such as the Pittsburgh Sleep Quality Index and sleep diaries [7]. Objective sleep measurements are assessed using behavioral and physiological technology, and thus are generally outside the participant’s control regarding the data collected [7]. Both objective and subjective measures tap into different parts of the sleep experience. For example, several studies have shown modest to null correlations between objective and subjective assessments of the same dimension [8-10]. Therefore, collecting both can provide a more complete picture of how sleep is affected by engaging in mindfulness meditation.

### mHealth Apps and Sleep

Research examining the impact of mHealth apps on meditation is in its early stages, with only a few randomized controlled trials testing the efficacy of these apps. Most of this work has been tested using the Calm app. For example, in a sample of adults with sleep disturbance, researchers found that Calm users had reduced self-reported daytime fatigue, sleepiness, and presleep arousal compared to a control group [11]. In a study among employees of a large retail company, researchers found that participants using the Calm app reported decreased daytime sleepiness compared to a control group [12]. Cross-sectional studies indicate that individuals who use Calm report longer and better sleep than those who do not use the app [13].

Yet, there is a dearth of knowledge about whether the Headspace app would also effectively improve sleep outcomes. Headspace is vital to study on its own, given its widespread use. For example, in September 2023 alone, Headspace was downloaded 400,000 times and generated $4 million in revenue [14]. Yet, while Calm is pitched as a multimodal app aimed at improving sleep using techniques that include meditation, Headspace operates from a different perspective. Namely, it was developed to provide step-by-step guidance on the basic principles of mindfulness meditation practice. In this way, Headspace serves as a mHealth corollary to the in-person Mindfulness-Based Stress Reduction training program. Indeed, research has demonstrated that the beneficial changes that occur directly from learning mindfulness meditation, such as decentering [15], acceptance [16], and nonreactivity to inner experience [17,18], are present after using Headspace [19]. As such, it is plausible that improvements in sleep may also ensue after using Headspace. Some initial evidence from quasi-experimental research has shown that children with attention-deficit hyperactivity disorder who used Headspace had reduced self-reported sleep disturbance after a 4-week intervention compared to baseline [20]. However, there is a need to rigorously investigate the impact of Headspace on various measures of sleep to further inform prospective users of its effectiveness.

Additional gaps exist in the current literature on mHealth meditation apps and sleep. Most research has focused on how mHealth meditation apps affect individuals with diagnosable sleep disorders. Yet, the usage rates of mHealth apps indicate that they are being used by the general population, who are likely not meeting clinical levels of sleep disturbance. Yet, poor sleep outcomes are still regularly reported among this population daily. For example, according to the 2020 Behavioral Risk Factor Surveillance System, 33.2% of adults report getting less than 7 hours of sleep per 24 hours [21]. Second, there is a need to test the effectiveness of Headspace using subjective and objective sleep measures to reduce the potential for expectancy effects to influence self-reports, which could account for significant effects. To this end, testing the effects of Headspace in daily life at several time points during the intervention can provide a richer picture of when mHealth apps affect sleep outcomes.

### The Present Study

This study tested whether the mHealth app Headspace affects several dimensions of sleep, including self-reported quality via a morning diary and daily tiredness via ecological momentary assessment (EMA), and objectively assessed duration and efficiency via a Fitbit Charge 2 wearable. We hypothesize that the use of a mHealth app will have a positive impact on both sleep quality and sleep duration. We also hypothesize that sleep quality and duration improvements will be recognized early on, possibly by the week 2 assessment. Finally, we do not have a clear hypothesis on how the improvements will last, as there is very little research on how sleep improvements change over time.

## Methods

### Participants

In this randomized controlled trial, participants were staff employees at a university in the San Joaquin Valley of California. Participants were excluded if they were not university employees or if they were university faculty, younger than 18 years old, not fluent in English, did not have access to a smartphone, or had prior experience in meditation, defined as having participated in mindfulness meditation 2 times per week for 10 minutes over the previous 3 months. To determine the sample size, we conducted a power analysis to compare 2 groups, assuming a moderate effect size, an alpha of .05, and a power of 0.80. The moderate effect size was used, given prior research suggesting such effects could be observed with this kind of mHealth app [22]. The power analysis results indicated that the required sample size to detect the effect was 128 participants. We aimed to enroll 140 participants to account for potential dropouts.

### Procedure

Participants were recruited through posted flyers on campus, presentations at departmental and university staff assembly meetings, and word-of-mouth referrals. Interested participants were directed to a secure website, where they read more about the study and were directed to a screener delivered through Qualtrics.

Participants attended an in-lab orientation lasting about 60 minutes. This orientation introduced participants to the assessment procedure. First, they downloaded the RealLife Exp app (Life Data Corporation) on their smartphone and loaded the survey. Participants were guided through a start-up session that demonstrated how to navigate the app and view examples of each question they would encounter during the study. Participants practiced how to answer and were able to ask questions about the process. After practicing, participants were instructed that when a survey was ready, it would appear as a push notification.

To assess subjective sleep quality, participants completed a daily diary upon waking. The survey was available for completion by 6:00 AM and remained open for 4 hours. Participants completed an EMA protocol throughout the day to assess tiredness. EMA surveys were completed randomly within each block: 8:00-10:00 AM, 10:30 AM-12:30 PM, 1:00-3:00 PM, 3:30-5:30 PM, and 6:00-8:00 PM. If participants did not immediately respond to a survey, they had up to 1 hour to complete it, with a reminder notification 20 minutes after the initial prompt. Surveys took between 3 and 4 minutes to complete on average.

Four consecutive days of data were collected via daily diary (quality) and EMA (tiredness). Each orientation took place on a Monday, Tuesday, or Wednesday morning. All data were then scheduled to begin data collection on Wednesday to ensure relative commonality across participants and to ensure both working (Wednesday through Friday) and nonworking (Saturday) days. Participants completed the 4 days of data collection (daily diary and EMA) as part of the baseline session and then at 2, 5, and 8 weeks after the first day of data collection at baseline.

Until this point, the researcher and participant were masked as to their condition. After randomization, both the researcher and the participant were unmasked for the duration of the study. Participants needed to be unmasked as they were instructed to engage with the Headspace app or not. Although researchers were also unmasked, they did not interact with participants outside of standardized messages sent during the study. Per instructions described during the in-lab orientation, participants were only randomized into the Headspace or control condition after the initial 4 days of data collection. Thus, weeks 2, 5, and 8 of data are postrandomization. Allocation to condition occurred at a 2:1 ratio, with more people in the Headspace condition, to account for the potential higher dropout rate in the intervention group as observed in mHealth studies [23]. A random number generator in Microsoft Excel was used to develop the order in which participants would be randomly allocated to a condition. To mimic the experience participants would normally encounter when downloading Headspace, minimal training was provided to participants by research staff. Participants in the Headspace condition were sent an email with download instructions for the app and a code to enter that would grant 12 months of access to Headspace. Headspace provided all the codes but had no say over data collection procedures, analyses, or dissemination. Participants were instructed to use Headspace for 10 minutes daily for the 8-week intervention period. The instructions specified that participants should complete each of the 3 Basic packs (with 10 units each) and then complete the Stress pack (with 30 units). To ensure compliance, we tracked downloads and initial use of the app, ensuring that all participants completed their first session within the first week postrandomization.

For sleep quality assessed via diary, at week 0, there were 462 assessments (mean 4.90, SD 0.56), 418 assessments at week 2 (mean 4.93, SD 0.44), 330 assessments at week 5 (mean 4.85, SD 0.76), and 237 assessments at week 8 (mean 4.85, SD 0.74). For tiredness assessed via EMA, at week 0, there were 2556 assessments (mean 18.66, SD 2.82), 2256 assessments at week 2 (mean 18.05, SD 3.83), 1832 assessments at week 5 (mean 18.87, SD 3.40), and 1326 assessments at week 8 (mean 18.46, SD 4.45). For sleep duration and efficiency assessed via Fitbit at week 0, there were 681 assessments (mean 4.80, SD 0.83), 487 assessments at week 2 (mean 4.87, SD 0.63), 315 assessments at week 5 (mean 4.50, SD 1.32), and 159 assessments at week 8 (mean 4.68, SD 1.17).

Participants in the control condition were given a 1-year subscription to Headspace after completing the 4-month waitlist period. During these 4 months on the waitlist, they were asked not to participate in any mindfulness activities such as yoga or meditation during this time. They completed the same daily diary and EMA protocol as participants in the Headspace condition.

### Measures

#### Baseline Measures

During the baseline assessment, participants first completed demographic information, including their gender (coded for analysis as 0=male, 1=female), age (in years), and ethnicity (coded for analysis as 0=non-Hispanic and Latino, 1=Hispanic and Latino), as well as other measures not relevant to this study.

#### Daily Diary and EMA Measures

Subjective sleep quality and tiredness were measured using EMA, based on methods from previous research that used EMA to assess physical well-being [24] and developed a standard daily sleep diary [25]. To assess subjective sleep quality, each morning, participants were asked, “How well did you sleep?,” on a scale from 0 (not at all well) to 10 (extremely well). To assess subjective tiredness, participants were asked, “Right now, how tired do you feel?”, rated from 0 (not at all) to 10 (extremely).

#### Fitbit

A Fitbit Charge 2 wearable was used to estimate sleep duration and efficiency. Fitbit is a popular fitness tracker with a microelectronic triaxial accelerometer to capture body motion in 3-dimensional space. These motion data are then analyzed using proprietary algorithms to identify motion patterns. The Fitbit Charge 2 also monitors heart rate activity through a patented photoplethysmography technology called PurePulse. PurePulse uses light-emitting diodes on the skin-facing surface to continuously estimate heart rate by monitoring blood volume changes [26]. Fitbit estimates steps, calories burned, and sleep through the estimated heart rate. Participants received training on the proper use of the Fitbit and the Fitbit app (Google LLC). To standardize this, participants were asked to wear the device continuously (including sleep) throughout the study. In addition, they were instructed to sync and charge it on the same days, Tuesday and Sunday, and to ensure the device was placed back on their wrist by the evening of each of these days. Fitabase (Small Steps Labs LLC) was used to extract and process the Fitbit data for the study. Fitabase measures duration as the total number of minutes asleep. Fitabase estimates efficiency by dividing the total time in bed by the total number of minutes asleep. To ensure data were valid, any sleep duration that was less than 2 hours or more than 12 hours was deleted from analyses (although patterns are similar when either or both criteria are not applied), as well as any efficiency scores greater than 100.

#### Headspace

The intervention was delivered through the commercially available mindfulness meditation app Headspace, widely used in previous intervention studies [27,28]. Headspace provides a variety of formal guided and unguided meditation practices, with instructions delivered through short, animated training videos. The intervention group was instructed to start meditating using the Basic pack. This pack is designed as an introduction to mindfulness meditation and can be used as an opportunity for participants to get familiar with the Headspace teaching style. Each lesson in the Basic pack is approximately 11 minutes and is led by a teacher of the user’s choosing. In the first part of the session, the teacher gives the user a short tutorial on the concept of mindfulness. Then, the teacher proceeds with a guided meditation. There are 3 total Basic packs, and each one has 10 sessions, with each session lasting approximately 11 minutes. If participants follow the directions of the study, they will finish all the Basic packs within 30 days. Once participants completed the Basic, they were instructed to move on to the Stress pack, which lasted for 30 sessions. Like the Basic pack, the Stress pack used a teacher and combined visualization and body scanning to help users learn to accept their emotions and pay close attention to the present moment.

Participants were encouraged to complete the meditation quietly for each session without distraction. They were instructed to use the app to meditate for 10 minutes a day for 8 weeks. This duration was chosen based on prior work, which showed that just 10 days of practicing mindfulness for 10 minutes a day successfully reduced stress, negative affect, and improved well-being among a range of sample types [29-32].

### Analytic Plan

We used multilevel modeling to account for the 3-level structure of our data, with multiple daily diary and EMA observations nested within assessment sessions (0, 2, 5, or 8) nested within participants. Models were tested using the PROC MIXED command in SAS v.9.4 (SAS Institute Inc), which tests a linear mixed model analysis. We used restricted maximum likelihood estimates to handle missing data, which is the recommended approach given its robustness in addressing missing data that is often nested (eg, a participant who dropped out in session 8 will have missing data for all diary and EMA observations) [33]. This procedure does not impute missing data but uses available data to calculate maximum likelihood estimates.

Analyses followed an intention-to-treat approach, assuming those randomized to the condition completed the intervention material as instructed [34]. As such, we did not factor in specific measures of usage of Headspace in analyses (an issue we return to in the “Discussion”). To test our main research question, we included the following variables as predictors: session, condition (0=control group, 1=Headspace group), and the interaction of session by condition. In the presented results, the terms labeled session refer to the effects of the control condition at that session relative to baseline. The interaction term of session by condition refers to the effects of comparing the Headspace to the control group at that session relative to baseline. Sleep was the outcome variable; each dimension of sleep was tested in a separate model. The session was modeled as a categorical variable to interpret whether the sleep data from sessions 2, 5, and 8 were statistically different than the session 0 data.

Finally, at the between-person level, models controlled for gender and age, and at the within-person level, models controlled for the day of the week as either a nonweekend (0) or weekend (1) day.

Ethical Considerations

The local Institutional Review Board approved all the study procedures and measures (IRB #UCM2018). This study was also registered on clinicaltrials.gov (NCT03652168). There were no deviations from the preregistered protocol, and the analyses presented are those for the secondary set of outcomes proposed. Data from the primary set of outcomes testing the effect of Headspace on mechanisms of mindfulness have previously been reported [19]. The participant completed informed consent at the initial intake session. Participants were reminded at all sessions that they could refrain from answering any questions they wished or could opt out of study procedures without penalty. All participants were given a study code to allow deidentification in the datasets. As compensation, participants received a 1-year subscription to Headspace. For each weekly survey (weeks 0, 2, 5, and 8), participants received US $15. In addition, participants could receive up to a US $20 bonus for a high completion rate (ie, over 80% of surveys completed) across the study.

## Results

Table 1 provides descriptive statistics for demographics and the sleep variables. The final sample (N=135) was between the ages of 21 and 65 years old and self-identified as primarily White or Hispanic, and female. Participants in the control condition (mean 40.88, SD 10.84, *t*_137_= 2.02; *P*=.05) were slightly older than participants in the Headspace condition (mean 36.91, SD 10.84, *t*_137_=2.02; *P*=.05). There were similar proportions of participants identified as Hispanic and female across the 2 conditions, *χ*^2^=2.29; *P*=.13, and *χ*^2^=0.28; *P*=.60, respectively. On average, participants reported sleeping moderately well each morning and feeling somewhat tired. They were recorded as sleeping about 5 and a half hours a night, and their sleep efficiency was below normal.

**Table 1.** Descriptive statistics for sleep outcomes.

Variable	Headspace group	Control group	Overall
Age (years), mean (SD)	36.91 (10.84)	41.12 (10.71)	38.19 (10.94)
Ethnicity, n (%)			
American Indian or Alaska Native	1 (1.04)	2 (4.76)	3 (2.17)
Asian	7 (7.29)	4 (9.52)	11 (7.97)
Black	3 (3.13)	1 (2.38)	4 (2.90)
Hispanic	30 (31.25)	8 (19.05)	38 (27.54)
Pacific Islander	2 (2.08)	1 (2.28)	3 (2.17)
White	57 (59.38)	26 (61.90)	83 (60.14)
Gender, n (%)			
Women	74 (77.08)	30 (71.43)	104 (75.36)
Men	22 (22.92)	12 (28.57)	34 (24.64)
Sleep quality, mean (SD)	5.87 (1.40)	5.31 (1.29)	5.68 (1.40)
Tiredness, mean (SD)	4.08 (1.65)	4.51 (1.44)	4.19 (1.56)
Total minutes asleep, mean (SD)	329.54 (63.70)	312.28 (44.34)	322.78 (71.52)
Efficiency, mean (SD)	74.74 (9.33)	75.03 (8.30)	73.93 (10.32)
Total time in bed, mean (SD)	449.36 (50.51)	417.13 (57.22)	447.70 (70.22)

**Figure 1.**
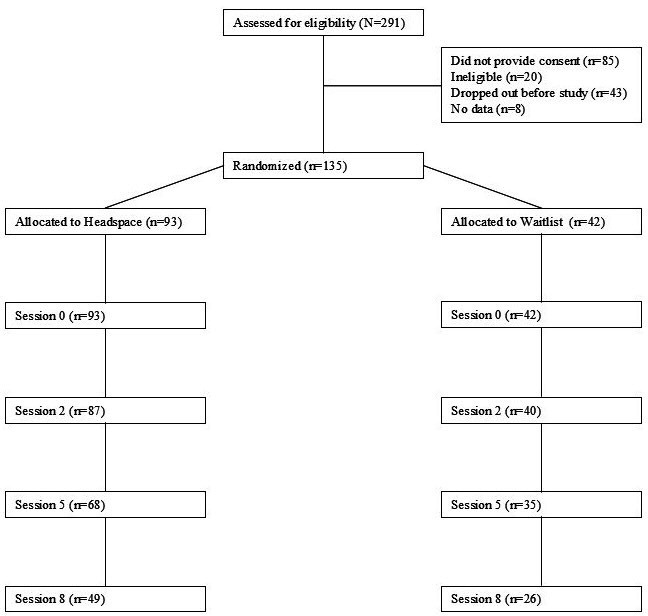
CONSORT (Consolidated Standards of Reporting Trials) diagram of participant flow.

Figure 1 provides a diagram of the process used to determine the final participant number for the study. An initial 291 employees were screened, with 271 eligible. Of the 271 eligible participants, 186 people provided informed consent. Before the study began, 43 participants dropped out, mainly citing time demands. One hundred forty-three participants were randomized, but 8 additional participants were dropped for not having any data. Randomization was completed using a random number generator via Excel to create the order in which participants would be enrolled and allocated to a condition.

At week 0, our sample consisted of 135 participants, with 93 randomized into the Headspace group and 42 into the waitlist control group using a planned 2:1 allocation strategy. At week 2, 87 (93.5% of the original Headspace group) and 40 (95.2% of the original control group) participants completed at least one daily diary and EMA survey. At week 5, 68 (71.5% of the original Headspace group) and 35 (83.3% of the original control group) participants completed at least one daily diary and EMA survey. At week 8, 49 (54.3% of the original Headspace group) and 26 (61.9% of the original control group) participants completed at least one daily diary and EMA survey.

We tested whether participants in the Headspace condition experienced improved subjective and objective sleep outcomes compared to those in the control condition, examining when these effects emerged over the 8-week intervention period.

We tested quality and tiredness for the subjective sleep outcomes with results reported in Table 2. The effects for the control group over time are represented as the session terms, whereas the effects for the Headspace condition compared to the control group over time are represented as session x Headspace terms. For quality, compared to the baseline session, those in the control condition reported worse sleep at session 5 (*P*<.001), with no differences at sessions 2 (*P*=.41) or 8 (*P*=.99). There were significant interaction effects of condition by session. Those in the Headspace condition, compared to the control group, reported better sleep at sessions 2 (*P*<.001), 5 (*P*<.001), and 8 (*P*<.001), with each session compared to baseline. For tiredness, those in the control condition reported, compared to the baseline session, an increase in tiredness at session 5 (*P*=.04), but not at sessions 2 (*P*=.85) or 8 (*P*=.79). There was a significant interaction effect of condition by session for session 5. Compared to the control group, those in the Headspace condition reported a decrease in tiredness at session 5 (*P*=.001), but no difference at sessions 2 (*P*=.97) or 8 (*P*=.97), with each session compared to baseline.

**Table 2.** Unstandardized beta estimates (SEs) for subjective sleep outcomes with Headspace, session, and interaction as predictors.

Subjective sleep outcomes	Subjective quality		Subjective tiredness	
	β (SE)	*P* value	β (SE)	*P* value
Random effects				
Intercept	1.63^a^ (0.21)^a^	<.001	2.29^a^ (0.30^a^)	<.001
Residual	3.33^a^ (0.06)^a^	<.001	4.41^a^ (0.09^a^)	<.001
Fixed effects				
Intercept	5.08^a^ (0.51^a^)	<.001	4.76^a^ (0.61^a^)	<.001
Female	−0.40 (0.26)	.12	−0.16 (0.31)	.61
Age	0.01 (0.01)	.18	−0.003 (0.01)	.79
Weekend	0.35^a^ (0.05^a^)	<.001	−0.32^a^ (0.07^a^)	<.001
Session 2	−0.08 (0.10)	.41	−0.03 (0.13)	.85
Session 5	−0.41^a^ (0.11^a^)	<.001	0.30^a^ (0.15^a^)	.04
Session 8	−0.002 (0.12)	.99	−0.04 (0.16)	.79
Headspace	0.24 (0.25)	.34	−0.34 (0.31)	.27
Session 2x Headspace	0.48^a^ (0.12^a^)	<.001	0.01 (0.16)	.97
Session 5x Headspace	0.91^a^ (0.13^a^)	<.001	−0.58^a^ (0.18^a^)	.001
Session 8x Headspace	0.69^a^ (0.15^a^)	<.001	0.01 (0.19)	.97
Model effects				
Pseudo *r^2^*	0.034	—^b^	0.017	—

a Coefficients significant at *P*<.05.

b Not applicable.

For the objective sleep outcomes, we tested duration and efficiency with results reported in Table 3. For duration, compared to the baseline session, those in the control condition had shorter sleep durations at session 5 (*P*=.03) compared to baseline, but not at sessions 2 (*P*=.94) or 8 (*P*=.20). There was a significant interaction effect of condition by session for session 5. Compared to the control group, those in the Headspace condition had longer sleep durations at session 5 (*P*=.04), but no difference at sessions 2 (*P*=.59) or 8 (*P*=.88), with each session compared to baseline. For sleep efficiency, compared to the baseline session, those in the control condition had a higher efficiency at sessions 2 (*P*=.04) and 5 (*P*=.002) compared to baseline, but not at session 8 (*P*=.33). There were no significant interaction effects of condition by session for sessions 2 (*P*=.37), 5 (*P*=.36), and 8 (*P*=.11).

**Table 3.** Unstandardized beta estimates (SEs) for objective sleep outcomes with Headspace, session, and interaction as predictors.

Objective sleep outcomes	Objective duration		Objective efficiency	
	β (SE)	*P* value	β (SE)	*P* value
Random effects				
Intercept	2404.70^a^ (571.52^a^)	<.001	4.19^a^ (0.99^a^)	<.001
Residual	4011.98^a^ (151.83^a^)	<.001	9.95^a^ (0.41^a^)	<.001
Fixed effects				
Intercept	353.93^a^ (32.26^a^)	<.001	89.70^a^ (1.39^a^)	<.001
Female	29.27 (15.04)	.05	0.80 (0.65)	.21
Age	0.57 (0.67)	.40	−0.04 (0.03)	.12
Weekend	11.20^a^ (4.12^a^)	.007	−0.37 (0.23)	.11
Session 2	−0.59 (8.16)	.94	0.98^a^ (0.46^a^)	.04
Session 5	−23.27^a^ (10.55^a^)	.03	1.77^a^ (0.58^a^)	.002
Session 8	−14.37 (11.21)	.20	0.59 (0.60)	.33
Headspace	23.32 (16.81)	.17	−0.69 (0.74)	.35
Session 2x Headspace	−5.26 (9.68)	.59	−0.49 (0.54)	.37
Session 5x Headspace	23.96^a^ (12.19^a^)	.04	−0.61 (0.66)	.36
Session 8x Headspace	2.12 (14.58)	.88	1.26 (0.79)	.11
Model effects				
Pseudo *r^2^*	0.058	—^b^	0.050	—

a Coefficients significant at *P*<.05.

b Not applicable.

## Discussion

### Principal Findings

This study used daily diaries, EMA, and Fitbit wearables to investigate the impact of Headspace on several dimensions of sleep. Our findings indicate that Headspace had the most significant effect on sleep quality, with some impact on tiredness and sleep duration, and no significant effects on sleep efficiency. Notably, improvements in subjective sleep (quality and tiredness) and objective sleep outcomes (duration) did not occur simultaneously but rather at different times during the study. These findings suggest the continued viability of using mHealth meditation apps to improve sleep and the likelihood that subjective and objective benefits may not happen in tandem. Furthermore, they are consistent with previous research on the Calm app [11,13] that illustrates sleep is multidimensional, with some dimensions improving while others fail to reach significance.

### Sleep Quality

The most consistent finding of this study was the rapid improvement in sleep quality. Participants reported better sleep quality within 2 weeks of using Headspace. This finding is impactful because there is emerging evidence that sleep quality is considered a superior sleep index to sleep quantity for assessing sleep [35]. Getting enough sleep is crucial for psychological and physical health, as there are negative consequences when sleep duration decreases past a certain amount of time [36]. What is interesting about sleep quality is that several studies have demonstrated that even if a person gets a whole night’s sleep, they can still report their sleep quality as poor and say they were unsatisfied with the night’s sleep [37-39]. These findings suggest that objective sleep measures do not fully account for subjective sleep quality. Studies have shown that sleep quality is a good indicator of psychological and overall health [40-42]. This also demonstrates that a person’s perceived efficacy of an intervention (which we suggest is as essential a dimension as objective measures) is a significant component that impacts adherence to the intervention [43]. As a future research direction, testing how the perceived efficacy of Headspace impacts adherence is of specific interest.

The improvements in sleep quality continued throughout the study. This promising finding shows that Headspace can alter a person’s thinking outside of novel effects. This may not seem profound, but if one considers that it is not uncommon for a person to claim an intervention changed them, then to only renounce that claim quickly thereafter. This is an example of the novelty effect, a well-known phenomenon further discussed below [44-46]. The fact that Headspace users reported continued improvements in sleep quality for 8 weeks indicates that this finding is more than likely not because of novelty and that Headspace does alter a person’s quality of sleep positively. This study did not investigate possible mechanisms; however, it has been suggested that meditation may improve sleep quality by reducing sleep-interfering cognitive processes [47], altering sleep architecture [48], changing perceptions of stress [49], and morphometric and connectivity alterations in sleep-related brain regions [50,51]. Taken together, these findings raise many unanswered questions, fueling future investigation.

### Intervention Length

A surprising finding is the convergence of improvements that peaked at week 5 in both subjective measurements, sleep quality and tiredness, and in one of the objective measurements, duration. This finding is novel in suggesting that there may be a previously unidentified point in time at which effectiveness peaks. It has been shown that 8 weeks, but not 4 weeks, of meditation is needed to see benefits in sleep quality and mood [52]. Equally, in non–sleep-related studies, 8 weeks of meditation increased brain structure and function [53] and mental health in university students [54], while 12 weeks of meditation improved depression, anxiety, and stress [55]. However, these studies measure 1 time point or compare 2 time points and cannot accurately pinpoint when effects emerge.

Although the 5-week time point may seem brief, research has shown the potential of mindfulness to have an acute positive effect even after a single use [56]. By allowing users to engage in meditation precisely when needed, such as before bed on a stressful day, and to use it frequently throughout the day in small but frequent doses (thus allowing microdose training), mHealth apps may accelerate the accumulation process. Indeed, other work in this sample found changes to the mindfulness mechanisms of acceptance, attention, and nonreactivity in as little as 2 weeks [19]. More broadly, these findings show the benefit of this study design to test for effectiveness during the engagement with Headspace and not simply wait for a postintervention evaluation period. This approach could be expanded in future studies to determine, with greater detail, the acute and longer-term effects of Headspace in each dimension of sleep. In the long term, this additional information will hopefully replace the “do ten minutes a day of Headspace” with a more nuanced approach.

It was unexpected that Headspace’s effects appeared to wane after 5 weeks. As suggested by other research, it was assumed that sleep benefits would continue throughout the 8-week intervention [51]. A possible explanation for this waning could be that the improvement was a placebo effect. A recent study showed that a sham meditation could increase state mindfulness, mindful observing, state decentering, and mindful nonreacting, similar to an actual meditation condition [57]. Yet, simply chalking up findings to a placebo effect seems unsatisfying. The continuous measurement of sleep daily, within and across days, would suggest an incredibly robust and omnipresent effect. Moreover, it is unclear why the placebo would wear off after 5 weeks. Nevertheless, future research should continue to probe how expected benefits from meditation may prime certain short-term perceived benefits.

There is another, broader hypothesis that may better explain the post-5-week waning: the well-known phenomenon that user engagement with mHealth apps decreases over time. For example, among users measured, 53% uninstall the app within 30 days [58]. Although determining the exact reasons for waning or discontinuing is challenging, research has shown that the main reasons are a loss of interest or declining motivation, a lack of desired features, and the app not being fun [59,60]. Ultimately, the novelty effect significantly influences user abandonment; users may be drawn to the new app out of curiosity but lose interest once it wears off [44-46]. Finally, attrition is a common phenomenon in longitudinal studies, with the literature suggesting that rates from 30% to 70% are not uncommon [61,62].

Of concern is that this pattern of waning, discontinued use, or attrition was observed despite the initial robust effects. The ability of these apps to run autonomously allows for their incredible reach [63]. However, without continued user involvement and self-management, behavioral changes and health improvements tend to dissipate [64]. Therefore, there is a need to continue developing features and behavior change techniques that sustain usage levels for long-term maintenance. In a systematic review of sleep apps, feedback and monitoring were used most often as a behavior change technique [65]. Yet, a recent meta-analysis identified 7 main areas that impact user engagement, with personalization—the ability to make technology act in a particular way depending on user preferences—being the element that users cited as most influencing their engagement [66]. Thus, there may be a disconnect between what app developers pursue as features to implement and what techniques may benefit long-term outcomes. App designers often focus on usability and acceptability rather than effectiveness in improving health outcomes [6]. Due to the increasing shift toward the use of mHealth apps and the vast number of apps available, user engagement is likely to become more critical as app developers strive to attract users. This will further the field of behavior change techniques and is a possible direction for future research.

### Limitations and Future Directions

A limitation of this study is that we were unable to track Headspace usage accurately and reliably. Therefore, we have yet to determine whether people in the Headspace group were meditating and are only relying on random assignments to the experimental condition to drive expected changes (ie, intention-to-treat analyses). In this way, we may be underestimating the effect of Headspace, as there may be nonusers in the Headspace group. In addition, tracking engagement would have allowed us to identify a type of person who was more likely to continue completing the intervention and to tailor the intervention content better to these individuals. Research from clinical psychology has shown that younger age, lower education, poorer social problem-solving, lower levels of persistence, and greater avoidance coping are factors of noncompletion [67]. Although not all these factors apply to the current intervention, it demonstrates distinct factors when examining noncompletion and why it is essential to track engagement.

As with most mHealth experimental research, attrition was a limitation, which may have led to the selection of certain traits related to study completion and sleep behaviors. For example, a recent meta-analysis found that the pooled attrition rate in young adults across 15 mHealth trials was 26% [67], and another found a pooled attrition rate of 43% [22]. This study’s attrition rate aligns with the attrition rates identified in these meta-analyses. Therefore, resolving or limiting attrition remains an unresolved issue, and addressing this would greatly benefit research aimed at determining interventions to reduce attrition.

### Conclusion

This study demonstrated that Headspace can improve sleep, showing that sleep dimensions do not all change with the same amount of meditation. Some sleep dimensions improved quickly, while others took longer to change. There was a significant convergence of benefits at the 5-week time point and increased attrition. These convergences uncovered 2 critical pieces of information: that there might be a lower-than-expected threshold of meditation needed to experience sleep benefits and that future research using mHealth apps must include factors that improve adherence and engagement. The current study showed that mHealth apps, specifically Headspace, are viable for those seeking to enhance sleep with minimal life disruption. The results from this study can be used by future scientists wanting to learn how to dose mHealth meditation interventions better and practitioners looking to use mHealth meditation to improve patient sleep outcomes.

## Supplementary material

10.2196/56287 Checklist 1 CONSORT checklist.

## Abbreviations

CONSORT: Consolidated Standards of Reporting Trials
EMA: ecological momentary assessment
mHealth: mobile health
